# Treatment of degenerative lumbar spine diseases with percutaneous PEEK rod and UBE: Four case reports

**DOI:** 10.1097/MD.0000000000043406

**Published:** 2025-07-18

**Authors:** Shuai Wang, Junling Pan, Xiaoxuan Zhuang, Zhengqi Chang

**Affiliations:** a Department of Orthopaedics, The 960th Hospital of PLA, Jinan, China; b Department of Reproductive Medicine, The 960th Hospital of PLA, Jinan, China; c University of Leicester Joint Institute, Chongqing Medical University, Chongqing, China.

**Keywords:** fat infiltration rate, lumbar degenerative diseases, PEEK rod, percutaneous screw fixation, unlateral biportal endoscopic

## Abstract

**Rationale:**

Traditional posterior lumbar fusion surgery can cause long-term complications such as muscle atrophy and adjacent segment degeneration due to the damage to the paraspinal muscles and rigid fixation.

**Patient concerns:**

The 4 patients in this case report all presented with the chief complaint of low back pain accompanied by lower limb pain.

**Diagnoses:**

The patient was diagnosed with lumbar degenerative diseases through clinical manifestations, imaging examinations, and physical examinations, including lumbar disc herniation, lumbar instability, and lumbar spondylolisthesis.

**Interventions:**

A retrospective analysis was conducted on the clinical data of 4 patients who underwent percutaneous polyether ether ketone (PEEK) rod pedicle screw fixation system combined with unilateral biportal endoscopic technique for lumbar degenerative diseases in our hospital from May 2022 to September 2022. Surgical data, imaging changes, and follow-up results were statistically analyzed.

**Outcomes:**

All surgeries were successfully completed, with all 4 patients undergoing single-segment fixation. The mean operation time was 162.50 ± 27.54 minutes, intraoperative blood loss was 70±24.49 mL, average bed rest time was 2.25 days, and average hospital stay was 5.5 days. The Visual Analogue Scale scores for low back pain and leg pain and the Oswestry Disability Index of the patients showed a gradually decreasing trend after surgery, and were significantly improved at the last follow-up compared with those before surgery, with statistically significant differences (*P* < .05). There were no significant changes in the cross-sectional area of the multifidus muscle, fat infiltration rate, range of motion of the operated segment, and disc height index at the last follow-up compared to preoperative values (*P* > .05). No complications, such as screw loosening or PEEK rod breakage occurred at the last follow-up.

**Lessons:**

Percutaneous PEEK rod pedicle screw fixation system combined with unilateral biportal endoscopic technique is feasible for the treatment of lumbar degenerative diseases.

## 1. Introduction

With the increasing aging population, the incidence of degenerative diseases of the lumbar spine is rising year by year, seriously affecting people’s quality of life.^[1]^ For patients with severe symptoms and ineffective conservative treatment, surgical treatment is often necessary.^[2]^ Spinal fusion surgery is widely used globally, and the number of surgeries is rapidly increasing. The classic procedure for treating degenerative diseases of the lumbar spine is the use of interbody fusion devices combined with posterior pedicle screw fixation systems. However, this surgical approach widely detaches the paraspinal muscles, leading to muscle atrophy, spinal instability, chronic pain, and even surgical failure.^[3]^ In addition, the titanium rod pedicle screw fixation system alters the normal mechanical conduction of the spine, increasing stress on the internal fixation system. At the same time, reducing the range of motion (ROM) of the fixed segment requires compensation from adjacent segments, increasing stress on neighboring segments, and leading to adjacent segment degeneration (ASD).^[4]^

Currently, there are 2 main challenges in traditional posterior lumbar fusion surgery: excessive trauma to the paraspinal muscles and persistent long-term complications stemming from rigid fixation. The unilateral biportal endoscopy (UBE) technique utilizes 2 separate channels for observation and operation, allowing for precise decompression in a clear and magnified view while minimizing damage to surrounding muscle tissue.^[5]^ The use of polyether ether ketone (PEEK) rods can reduce the incidence of ASD and maintain spinal mobility.^[6]^ Studies have shown that dynamic internal fixation with PEEK rods preserves a greater ROM in the fixed segment compared to titanium rod fixation systems, particularly in axial rotation, lateral bending, and flexion.^[7]^

In response to the challenges and recent advancements in the field, our department launched a study in May 2022 focusing on the efficacy of a percutaneous PEEK rod pedicle screw internal fixation system combined with UBE technology for the treatment of lumbar degenerative diseases. This article examines data from 4 patients to explore the clinical effectiveness of treating lumbar degenerative diseases, focusing on the preservation status of paraspinal muscles and the retention of patients’ quality of life. Fixed segment ROM and changes in intervertebral height.

## 2. Material and methods

### 2.1. General information

Inclusion criteria: patients with degenerative diseases of the lumbar spine, including lumbar disc herniation, lumbar spinal stenosis, and possible lumbar instability or isthmic spondylolisthesis; with symptoms of lower back pain, leg pain, or neurological symptoms that have not improved with nonsurgical treatment for more than 3 months. Exclusion criteria: spinal infections, fractures, tumors, scoliosis or kyphosis deformities; Meyerding grade ≥ II isthmic spondylolisthesis; severe osteoporosis.

**Figure 1.**
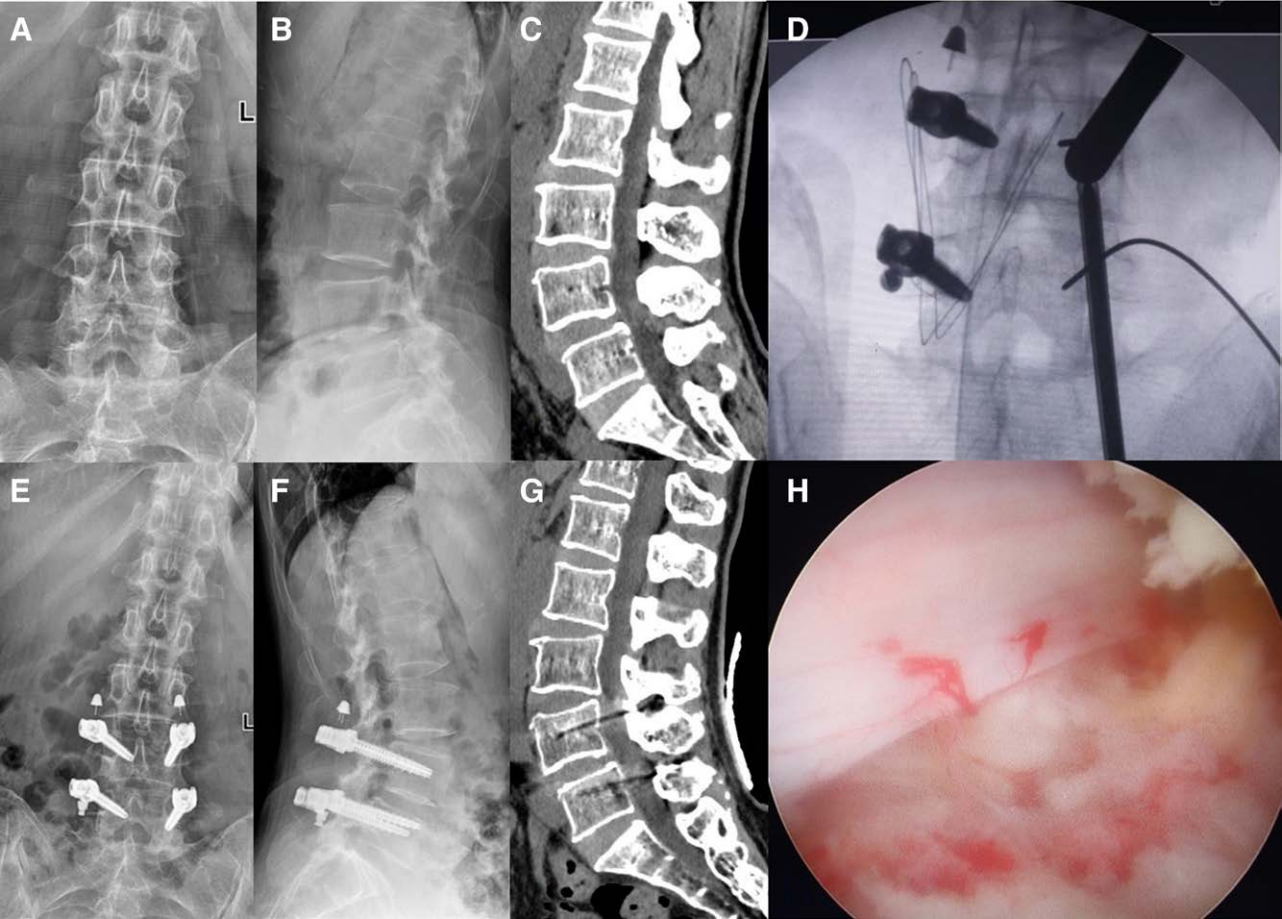
(A–C) Preoperative lumbar spine anteroposterior and lateral X-rays and lumbar spine CT sagittal images showing mild anterior slippage of the L4 vertebral body. (E–G) Postoperative follow-up lumbar spine anteroposterior and lateral X-rays and lumbar spine CT sagittal images showing good placement of screws and PEEK rod. (D) Left pedicle screw and mock rod inserted during surgery, right side secured with UBE working sleeve. (H) Nerve root photo taken after satisfactory decompression. CT = computerized tomography.

Based on the above criteria, this study analyzed the data of 4 patients who underwent percutaneous PEEK rod pedicle screw fixation system combined with UBE technique for the treatment of degenerative lumbar spine diseases at the PLA 960 Hospital from May 2022 to September 2022 (Table 1). This study has been approved by the 960th hospital of PLA. Each author certifies that all investigations were conducted in accordance with ethical principles. Informed consent was obtained from all individual participants in the study, who also provided written consent for the publication of this article. Proof of consent to publish from the study participants is available upon request. Preoperative evaluations included lumbar spine X-rays, flexion-extension views, computerized tomography scans, and magnetic resonance imaging (MRI) scans (Fig. 1A–C).

**Table 1:** The baseline of included 4 patients.

Items	Patient 1	Patient 2	Patient 3	Patient 4
Sex	Woman	Woman	Woman	Woman
Age (year)	54	67	46	65
BMI (kg/m^2^)	25.3	27.1	20.4	25.4
Smoking	No	No	No	No
Hypertension	No	No	No	Yes
Diagnosis	LDH + LI	LDH + LI	LS (I°)	LDH + LI
Levels	L5/S1	L4/5	L4/5	L5/S1
Operation time (min)	180	150	190	130
Time in bed (day)	2	2	3	2
Length of postoperative hospital stay (day)	7	6	4	5
Bleeding (mL)	100	50	80	50
Follow-up time (months)	6	6	7	6

LDH = lumbar disc herniation, LI = lumbar instability, LS = lumbar spondylolisthesis.

### 2.2. Procedure and postoperative care

All surgeries are performed by a dedicated team of doctors with extensive experience and a proven track record of successful procedures. After general anesthesia, the patient is placed in a prone position. Taking the example of the right L4/5 level, 4, 1 cm incisions are made at the outer edge of the bilateral lamina of L4/5. The skin is cut open to the fascia layer, and after accurate positioning, an appropriate length pedicle screw is inserted on the left side of L4/5 under the guidance of a guide wire. A suitable length trial rod is placed on the left side, and after gently spreading the intervertebral space, the set screw is pre-tightened. A UBE working sleeve is placed on the right side of L4/5 (Fig. 1D), and the upper part of the L5 inferior articular process and the inner part of the L4 superior articular process are removed. The ligamentum flavum is exposed, and a radiofrequency hole is made in the ligamentum flavum. The upper edge of the L5 vertebral plate and the lower edge of the L4 vertebral plate are removed, and a right L4/5 laminar gap is widened to decompress the right L5 nerve root. The right L5 nerve root is exposed, and a slight protrusion of the intervertebral disc is seen in front of the shoulder of the L5 nerve root. The protruding nucleus pulposus tissue is completely removed, and after thorough decompression (Fig. 1H), the appropriate length pedicle screw is inserted on the right side of L4/5 under the guidance of a guide wire (Fig. 1E–G). A PEEK rod is placed on the right side, and the set screw is tightened. The left trial rod is replaced with PEEK rod is inserted, and after verifying the satisfactory position and depth of internal fixation through C-arm fluoroscopy, the incisions are meticulously closed in layers, sterile dressings are applied, marking the completion of the surgery. Postoperatively, dehydration and nutritional nerve medication are given, and antibiotics are used for 24 to 48 hours to prevent infection. The patient is encouraged to get out of bed on the second day after surgery. Back muscle function exercises begin 1 week postoperatively, and a back support belt is worn for 1 to 3 months.

### 2.3. Information collected

**Figure 2.**
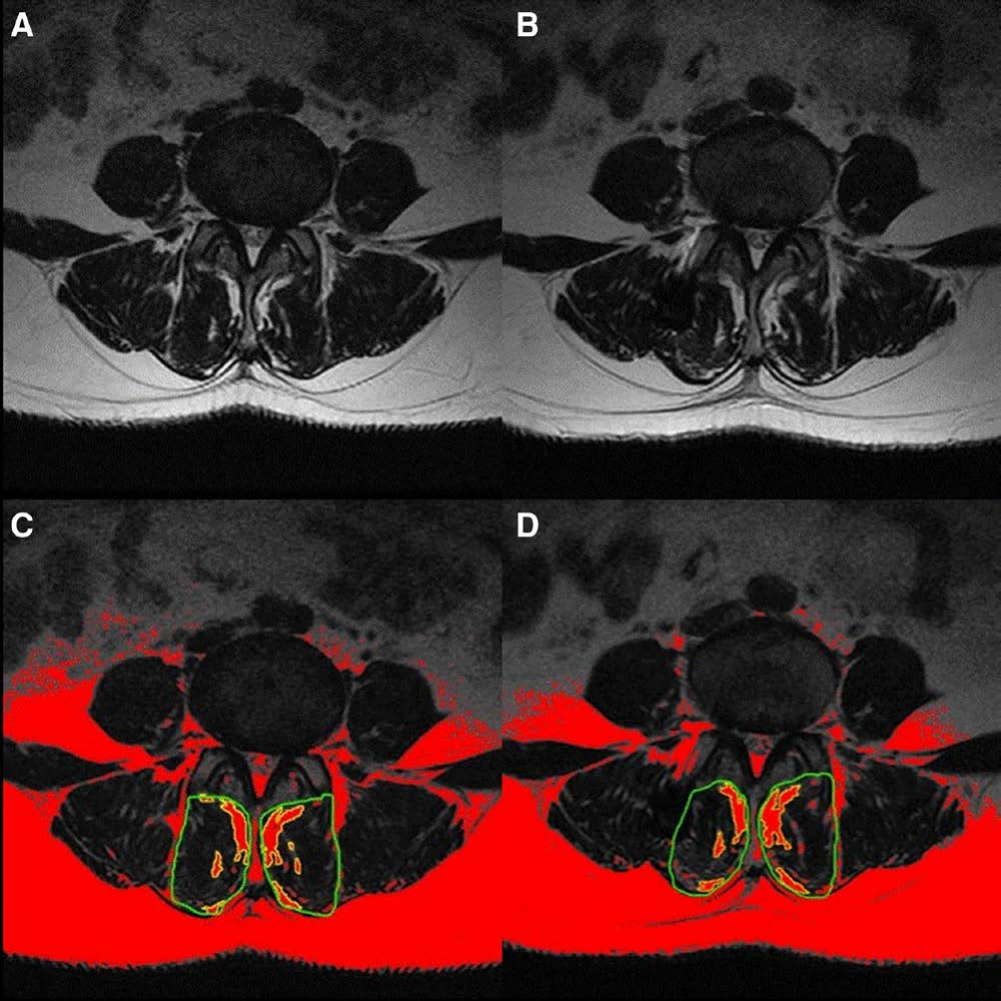
The Image J software was utilized to mark the multi-fissured muscle region (green border) in the selected MRI T2-weighted image, with red indicating adipose tissue and black-gray indicating muscle tissue. (A, C) MRI before surgery and images showing the measurement of fat infiltration rate. (B, D) MRI at 7-month follow-up after surgery and images showing the measurement of fat infiltration rate. MRI = magnetic resonance imaging.

Observation indicators include recording intraoperative blood loss, surgical time, bed rest time, and time from surgery to discharge, as well as documenting intraoperative and postoperative complications. Pain levels before and after surgery are assessed using the visual analogue scale (VAS) and functional disability is evaluated using the Oswestry Disability Index (ODI). Changes in disc height are evaluated by measuring the disc height index on lateral lumbar spine X-rays of disc height to the height of the superior vertebral body. The ROM of the surgical segment is measured on flexion-extension lumbar spine X-rays. The cross-sectional area of the bilateral multifidus muscles is measured on lumbar spine MRI scans to calculate muscle atrophy rate. The fatty infiltration rate of the multifidus muscles is calculated using Image J software to assess muscle quality (Fig. 2), and muscle degeneration is graded using the Goutallier classification. Perioperative complications are defined as complications occurring within 1 month postoperatively, such as wound infection, wound dehiscence, cerebrospinal fluid leakage, and fat liquefaction. Screw loosening is defined as the presence of ≥ 2 mm radiolucent lines on both sides of the screw, and screw fractures are observed on X-rays.

### 2.4. Statistical analysis

SPSS 26.0 software was utilized for the statistical analysis of the data. Normality tests were performed across all datasets. When the data met the criteria for normal distribution, quantitative data was presented as mean ± standard deviation (x ± s), with *t* tests employed for categorical data. For data that did not conform to normal distribution, quantitative data was reported as median with interquartile range (M (QR)), and nonparametric tests were applied to categorical data. The one-way repeated measures analysis of variance was used to analyze the changes in postoperative VAS scores and ODI. The significance level was set at α = 0.05, with *P* < .05 indicating statistically significant differences. The sample size of this study is relatively small, but this statistical method is still applicable.

## 3. Results

### 3.1. General information

All surgeries were successfully completed without any complications. The 4 patients were females with an average age of 58 (range 46–67) years. The surgeries had an average duration of (162.50 ± 27.54) minutes, and the average amount of blood loss during surgery was (70 ± 24.49) mL. The mean bed rest time was 2.25 days, and the average hospital stay was 5.5 days. Three patients had lumbar disc herniation, while 1 had lumbar spondylolisthesis. All 4 patients underwent single-segment fixation, with 2 cases at L4/5 and 2 cases at L5/S1, as outlined in Table 1. There were no postoperative complications reported.

### 3.2. Evaluation of clinical outcomes

**Figure 3.**
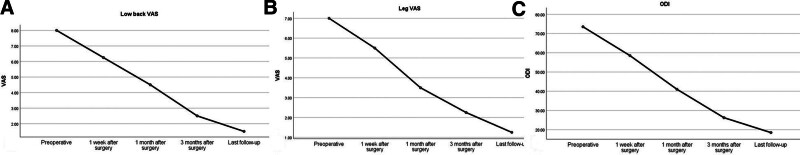
(A, B) Over time, the patient’s low back pain and leg pain have significantly improved. (C) Over time, the patient’s ODI decreased significantly. ODI = Oswestry Disability Index.

At the last follow-up, the patient’s VAS score for low back pain improved from 8 ± 0.82 preoperatively to 1.5 ± 0.58, and the VAS score for leg pain improved from 7 ± 0.82 to 1.25 ± 0.50. The ODI also improved from (74 ± 10)% to (19 ± 3)%, with statistically significant differences (*P* < .05), as shown in Table 2. The VAS scores for low back pain and leg pain and the ODI of the patients showed a gradually decreasing trend after surgery (*P* < .05), as shown in Figure 3.

**Table 2:** Preoperative and postoperative data were compared.

	Pre-op	Post-op	*T* value	*P*-value
Low back VAS	8 ± 0.82	1.5 ± 0.58	t = 10.070	.002
Leg VAS	7 ± 0.82	1.25 ± 0.50	t = 9.139	.003
ODI	(0.74 ± 0.10)%	(0.19 ± 0.03)%	t = 10.856	.002
CSA	49.96 ± 11.08	49.63 ± 9.78	t = 0.487	.659
FI	(20.11 ± 2.77)%	(19.70 ± 3.19)%	t = 0.880	.444
DHI	0.44 ± 0.08	0.45 ± 0.07	t = -1.464	.239
ROM	8.30 ± 9.67	8.11 ± 8.92	t = 0.448	.685

CSA = cross-sectional area, DHI = disc height index, FI = fatty infiltration, ODI = Oswestry Disability Index, ROM = range of motion, VAS = visual analogue scale.

### 3.3. Imaging data analysis

The preoperative cross-sectional area was 49.96 ± 11.08, and at the last follow-up it was 49.63 ± 9.78; the preoperative fatty infiltration rate was (20.11 ± 2.77)%, and at the last follow-up it was (19.70 ± 3.19)%; the preoperative disc height index was 0.44 ± 0.08, and at the last follow-up it was 0.45 ± 0.07; the preoperative ROM was 8.30 ± 9.67, and at the last follow-up it was 8.11 ± 8.92; there were no statistically significant differences in any of the data between the groups (*P* > .05), and all patients had Goutallier grade 2 at both preoperative and last follow-up, as shown in Table 2.

## 4. Discussion

Open traditional posterior lumbar fusion surgery is currently the classic surgery for treating degenerative lumbar diseases, but the destruction of the back muscles and ligament structures may lead to complications such as muscle atrophy and chronic lower back pain.^[8]^ Spinal stability is closely related to the paraspinal muscles, and the multifidus muscle plays a crucial role in maintaining lumbar stability and controlling lumbar movement due to its anatomical structure and morphology.^[9]^ The multifidus muscle, located on the innermost side of the spine, has the largest attachment area of the paraspinal muscles. It has a larger physiological cross-sectional area and shorter muscle fibers, with a rich content of muscle fibers in the muscle belly, allowing it to generate strong forces to maintain spinal stability.^[10]^ Functionally, the main purpose of multifidus muscle contraction is not to produce lumbar movement, but to resist spinal rotation and sliding, maintaining the lumbar spine’s anterior curvature and being an important factor in spinal dynamic stability.^[11]^ Therefore, excessive stripping of the paraspinal muscles during conventional posterior open surgery may not only result in large trauma and slow recovery but also lead to muscle atrophy and permanent loss of function.

In recent years, with the rapid development of minimally invasive concepts and techniques, endoscopic spine surgery has been widely used in clinical practice. The UBE technology, based on independent imaging and operating systems, creates a good working space for surgeons through continuous irrigation with physiological saline. Additionally, UBE technology can directly advance the surgical field to the vertebral plate or enter the contralateral lateral recess using an endoscope, achieving unilateral approach bilateral decompression.^[12]^ Particularly importantly, the UBE technique is carried out entirely under the endoscope, which preserves the integrity of the paravertebral muscles as much as possible and minimizes the damage to the multifidus muscle.^[13]^ Importantly, UBE technology can avoid stripping the paraspinal muscles, minimizing muscle damage. Muscle degeneration is mainly assessed by muscle volume reduction and increased fat deposition. In this study, we observed no significant changes in the cross-sectional area and fat infiltration rate of the multifidus muscles postoperatively using MRI. We believe that the minimal stripping of the paraspinal muscles during surgery prevents irreversible muscle damage, and early postoperative mobilization can prevent disuse muscle atrophy. Therefore, UBE technology has unique advantages in preserving paraspinal muscles and maintaining spinal stability.

Although UBE technology is better at preserving the facet joints compared to other spinal endoscopic surgeries, the inevitable facet joint resection during surgery may accelerate facet joint degeneration, leading to iatrogenic segmental instability.^[14,15]^ Research shows that PEEK rods can effectively reduce the bone–screw interface stress. The trabecular bone around the PEEK rod screws is denser and thicker than that around titanium rods, effectively reducing the risk of screw loosening.^[16,17]^ Additionally, PEEK material has good anti-infective properties, which is beneficial for preventing postoperative wound infections.^[18,19]^ To address potential instability caused by preoperative vertebral instability or intraoperative decompression-induced instability or loss of intervertebral height, we performed internal fixation using percutaneous PEEK rod pedicle screw technology to effectively compensate for the compromised stability of the spine structure, preserve the ROM of the fixed segments, and reduce the incidence of ASD. In this study, at the last follow-up, there was no significant change in the range of motion of the surgical segments compared to preoperatively. None of the patients developed adjacent segment degeneration, infection, or screw loosening.

Research shows that the postoperative symptom improvement rate of UBE is 81% to 88%, while that of traditional decompression surgery is only 64%.^[20,21]^ Compared with the minimally invasive transforaminal lumbar interbody fusion technique, the UBE technique has no significant difference in the incidence of postoperative complications, but it has a faster postoperative recovery, a shorter hospital stay, and a lower postoperative infection rate.^[22,23]^ To prevent the formation of deep venous thrombosis in the lower extremities post-surgery, particularly for high-risk patients, including the obese, anticoagulant drugs remain a standard practice following UBE surgery, albeit with a shorter application duration and reduced dosage, which is beneficial for reducing the risk of bleeding and the incidence of postoperative epidural hematoma formation.^[24,25]^ Percutaneous PEEK rod pedicle screw internal fixation and UBE technology are both minimally invasive procedures, and by combining the advantages of both, the study results show that the 4 patients exhibited characteristics of minimal trauma and minimal blood loss, consistent with previous literature reports.^[26,27]^

The deficiencies of this study are as follows. Firstly, centralized hospital procurement issues led to a shortage of PEEK rods, limiting the study’s case number. Secondly, the short follow-up time for patients may have impacted the results analysis. Currently, we have resolved the centralized procurement issue for PEEK rods and aim to conduct large-scale surgeries for this study. Furthermore, we plan for multi-center studies with larger samples, long-term follow-up studies are needed to provide more insights.

In conclusion, the percutaneous PEEK rod pedicle screw internal fixation system, when combined with UBE technology, has been shown to be a safe and minimally invasive treatment option for degenerative lumbar diseases. This approach not only reduces damage to the paraspinal muscles and adjacent lumbar segments but also maximizes the preservation of lumbar mobility, as evidenced by clinical studies.

## Author contributions

**Conceptualization:** Junling Pan, Zhengqi Chang.

**Data curation:** Shuai Wang, Junling Pan, Xiaoxuan Zhuang.

**Formal analysis:** Junling Pan, Zhengqi Chang.

**Investigation:** Shuai Wang, Junling Pan, Xiaoxuan Zhuang, Zhengqi Chang.

**Methodology:** Shuai Wang.

**Writing – original draft:** Shuai Wang, Junling Pan, Xiaoxuan Zhuang.

**Writing – review & editing:** Zhengqi Chang.
